# Blepharitis Ciliaris and Phlyctenular Conjunctivitis

**Published:** 1866-03

**Authors:** E. Williams

**Affiliations:** Professor of Ophthalmology, and Aural Surgery, Miami Medical College, Cincinnati, Ohio


### BLEPHARITIS CILIARIS AND PHLYCTENULAR CONJUNCTIVITIS.

By E. WILLIAMS, M.D., Professor of Ophthalmology, and Aural Surgery, Miami Medical College, Cincinnati, Ohio.

It is not my purpose to give a lengthy description of these common and well-known diseases, but to call your attention to some practical points in their treatment. Still a hasty sketch of their characteristic features will facilitate a better appreciation of the therapeutic measures which I wish to present. Whether associated, or existing separately, blepharitis ciliaris (sometimes called blepharitis marginalis—bleparadenitis—tinea tarsi) and phlyctenular or pustular conjunctivitis occur usually in early life and in the same class of patients. The period of life at which they are most frequent is from the second to the fifteenth year. If they are seen after that age, it will generally be found to be but a continuation of the same affection from an earlier period. Children of a strumous or scrofulous diathesis are the almost exclusive victims of these maladies.

Strictly speaking, blepharitis ciliaris, as the name intimates, commences in the individual follicles of the eyelashes, and invades the other textures of the lids subsequently. It is an inflammation of those follicles followed by little abscesses, of which the cilia form the centres. These minute collections of pus become confluent along the edge of the lid, agglutinating the lashes in little tufts, and forming scabs or crusts as they desiccate. These adhere very firmly, and when removed forcibly, produce pain, often bring away some of the hairs, and the surface beneath is found to be raw or ulcerated—perhaps bleeds. These patches of disease may be limited, or extend the entire length of the lid. When this local difficulty has lasted for some months or years, the bulbs of the lashes are gradually obliterated, the hairs fall out, grow in a stinted form, or cease altogether; giving rise to that thickened, red, perhaps partially everted state of the edge of the lid, called *lippitudo.*

Blepharitis ciliaris, when once well established, usually persists for years, or for the remainder of life if cleanliness and favorable hygienic measures are impracticable or not enforced. The amount of redness, swelling, and soreness of the lids varies very much under special provocations or favorable circumstances. The smouldering fire at the roots of the lashes, however, never ceases; and that is the focus from which they flame out. • Intercurrent attacks of phlyctenular conjunctivitis often complicate and aggravate the difficulty; and not very unfrequently, in the chronic forms, there is set up blennorrhœa of the lachrymal sac, with its consequences. On the contrary blepharitis marginalis may, in its turn, be provoked and perpetuated by dacryocystitis, stricture of the nasal duct, and dilatation of the sac. Now these conditions mutually aggravate each other, and the necessity of embracing them all in the treatment is self-evident. Eczema and other eruptions about the face and head, and especially in and around the nostrils, causing a constant running, with redness and thickening of the nose, or the formation of scabs, are likewise a very frequent and troublesome complication.

Phlyctenular conjunctivitis (often called scrofulous ophthalmia), is marked by symptoms so peculiar as not to require a tedious description. It is a partial inflammation, commencing in the conjunctiva of the globe, and attended by pustules or vesicles. These so-called phlyctenulæ are most commonly seated on the line of junction of the cornea and sclerotica, but sometimes they are seen entirely on the one or the other of these structures. They are inflammatory accumulations of serum or of pus under the epithelial layer of the conjunctiva, and when located on the cornea are designated phlyctenular keratitis. These may be absorbed without rupturing, but generally the elevated epithelium bursts and leaves superficial ulcers, called on the cornea, facetted ulcers. In favorable cases they then heal, by being first covered with a new epithelial layer, that makes them appear shiny and smooth in the bottom, and a subsequent reproduction of the deeper layers. On the cornea they leave little circular depressions, readily seen by looking obliquely at them, which persist a very long time before they are filled to a level with the general surface. If the ulcer extends deep into the lamellæ of that membrane, permanent, or at least long persisting, white specks or opacities are left behind. When situated towards the centre of the cornea, and a bundle of vessels develops under the epithelium, running from the conjunctiva scleroticæ to the pustule, lymph often exudes in a narrow strip along the course of the vessels, leaving a concentric opaque streak in the cornea. In some cases, on the other hand, a series of vesicles following from the margin towards the centre, are produced, attended by the gradual pushing forward of the little loops of vessels, and leaving when they subside the same riband-like opacity just mentioned.

So long as the phlyctenulæ are confined to the conjunctiva scleroticæ, there is seldom any marked intolerance of light or other suffering. But when they come out on the cornea, especially if near the centre, they nearly always give rise to extreme photophobia, profuse lachrymation, and sometimes severe pain. The intolerance of light is so intense often, that the little sufferers seek the darkest corners, lie on their faces and bury their eyes, covered with their hands, in a pillow or anything they can get, so as, if possible, to exclude every ray of light. This confines the perspiration, heats the eyes, aggravates the eruptions of the face, and is in all respects the most distressing symptom, lasting frequently for weeks together before any light can be endured. This feature of the disease is more marked in the morning, giving way towards evening, so that after sundown the child may open its eyes better. This excessive tenderness to light, resulting in reflex, spasmodic contraction of the orbicularis palpebrarum, and profuse lachrymation, is the result, in my judgment, exclusively of irritation of the corneal filaments of the fifth pair of nerves. Whenever I see these delicate, scrofulous children, with their eyes covered and spasmodically closed, I am certain that they have corneitis with little specks or ulcers. Still I always insist on holding the child's head between my knees, with its face up, and forcibly opening and examining the eyes, so as to see the exact state of the cornea. This is less cruel and also less *irritating to the mother*, than plunging the child's face in ice-water, as recommended by Graefe, and others.

In extreme cases chloroform may be used, but I seldom find it necessary. The photophobia is frequently out of all proportion to the amount of local trouble; but there is always some deposit in the cornea, or abrasion of its surface, which can be detected by a proper inspection.

It is comparatively seldom that these phlyctenulæ give rise to ulcers that invade the deeper layers of the cornea; but sometimes this occurs. The edges and bottom of the ulcer then become infiltrated with a dirty-white or yellowish-colored deposit, which, as it goes deeper and deeper, becomes complicated by hypopium, and eventually, if not properly treated, results in perforation and prolapsus of the iris. This tendency to deep ulceration and perforation of the cornea in pustular conjunctivitis, is sometimes epidemic. A few months ago, some seventy or eighty children in one of our neighboring orphan asylums, were attacked within a few days with this disease. Rapid ulceration, hypopium, and perforation took place in many of them, and several were blinded in one or both eyes before any treatment was thought of. Some of them had had granulated lids previously, and some had not. The affection assumed the form of general catarrhal conjunctivitis, with large sloughing phlyctenulæ, which were not influenced by anything but repeated paracenteses of the cornia, atropine, and compression with cotton and an elastic bandage.

In the treatment of this class of diseases I have used for the last eight years a preparation, not found in any of our books, which is so uniformly and promptly beneficial, that I have abandoned all the other mercurial local applications in its favor. When *opportunely* and properly applied, it acts like a charm, and patients or their friends nearly always come back, if they ever have another attack, and ask for some more of that *brown salve.* The formula for its preparation, I found in a foot-note in an old edition of Wilde on the Ear. He recommended its use very highly in chronic diseases of the dermoid lining of the meatus auditorius externus. It occurred to me that its peculiar properties might prove useful in the diseases under consideration. I tested it extensively and faithfully, and it has far surpassed my most sanguine expectations. I call it the *brown citrine ointment*, and write it Unguent. citrin. rub. At my request, Mr. A. Fennel and Prof. E. L. Wayne, well-known chemists and druggists of this city, have experimented in making the ointment till they have very much improved its quality. The original formula was the same as that of the U.S. Pharmacopœia for the preparation of citrine ointment, excepting that cod-liver oil was substituted for the axungia and the neatsfoot oil. Still it was somewhat granular and coarse, till Prof. Wayne finally succeeded by *reheating*, and then stirring till it was cool, in making a dark mahogany-brown salve of uniform consistence, tenacious, and readily melting with the temperature of the body. When rubbed on the lids or applied to the conjunctiva, it melts in a few seconds, spreads and adheres a long time to the lids, or, if used in the eye, is rapidly diffused over the ball by the movement of the lids, with *very little irritation.* It can be used in its pure state, and does not need to be *removed* afterwards at all. My treatment for blepharitis ciliaris is very simple. The first thing on which I always insist, and without which no treatment does much good, is to keep the edges of the lids *absolutely free from scabs or scales.* I have the eyes bathed with tepid water morning and evening, till the scabs are soft and can be readily scraped off, or rubbed off with a soft rag. If there is much rawness and soreness of the margins of the lids, I trim off the lashes with a fine pair of scissors, as close as possible. This is much better than pulling them out, as recommended by some authors; and when so trimmed, it is much easier to keep the lid free of scabs. If the lids are swollen and very sore, I have them poulticed every night for a few days; and sometimes when there are ulcers or raw places along the roots of the lashes, I touch them lightly for a few times, at intervals of two or three days, with a point of nitrate of silver. Every night, after the washing and cleaning, the salve is rubbed well along the edges of the lids with the finger. If it irritates and causes the skin to puff up and look redder next day, which it rarely does, the patient may omit the use of it the next night; otherwise, it should be applied every evening at bedtime. Its continuance two or three times a-week for several weeks or months after the disease is entirely relieved, should be urged, to prevent a relapse. If there is much conjunctivitis, I prescribe a solution of four grains of sulphate of morphia, and half a grain of sulphate of copper or zinc, to the ounce of water, dropped into the eye three times a-day. Internally, such patients should always take iron or tonics of some kind. I usually gave the liquor ferri iodidi, in doses suited to the age of the subject, and keep it up for several months, to secure a *radical result.* Of course, regular exercise in the fresh air, good nourishing diet, bathing followed by friction, and the avoidance, as far as possible, of all causes of irritation, are important hygienic measures in this class of diseases.

In phlyctenular conjunctivitis where the vesicles have their seat on the conjunctiva scleroticæ, or being on the cornea, give rise to but little intolerance of light and lachrymation, this salve again is most valuable. A portion of it, say a small drop, should be put from the end of a knitting-needle or probe, between the lower lid and the ball and the upper lid, then moved up and down a few times till it is spread freely over the cornea. This ought to be done at bedtime. The larger portion of the salve is soon washed and pressed out on the edges of the lids, and, if blepharitis exists, can be usefully rubbed along the margin; otherwise it may simply be wiped away. If the conjunctivitis is more general, and there is much mucous secretion, I direct the astringent given above; otherwise, the ointment alone suffices perfectly.

Phlyctenular corneitis is treated in the same manner, when the acute symptoms have begun to abate, or when it assumes a mild form from the start. If there is marked dread of light, spasmodic closure of the lids, and epiphora, a few days of preparatory treatment are necessary. For the relief of the photophobia and other symptoms arising from acute inflammation of the cornea, there is nothing equal to the local application of a solution of atropia. In a child from two to five years old, I prescribe a solution of gr. j. to aquæ ℥j., to be dropped into the eye from three to six times in the twenty-four hours, according to the intensity of the symptoms. If they are over five years old, a two-grain solution may be directed. Internally, the sulphate of quinine in very liberal doses, say from one to two grains, even more where the photophobia is extreme and obstinate, three times a-day, with the solution of sulphate of atropia locally, nearly always overcomes these distressing symptoms in a few days. If the quinine alone does not subdue the intolerance of light, I combine opium with it, giving from half a grain to a grain with the quinine three times a-day. Under this treatment, the child begins to open its eyes and bear the light better in a short time. It is astonishing what effect the atropia has in quieting the eye and allaying the inflammation. When the acute symptoms have decidedly abated under this treatment, the salve in the eyes once a-day may be tried, and if well borne, kept up for several weeks. It then not only allays irritation, and favors the healing of the phlyctenulæ, but promotes the absorption of the remaining opacities. After the first few weeks the quinine may be left off, or continued in smaller doses, in combination with iron; or, what is preferable, the iodide of iron may be substituted for it. When the acute symptoms and redness of the eyes are relieved, the atropin also may be suspended, and the case left to the use of the salve alone.

The eczema, and other eruptions about the face, should be smeared every night with the same ointment. It acts very promptly in relieving all scabby eruptions on the face, head, and elsewhere. Instead of fearing to heal these up, I always try to get rid of them—especially the sore nose, which keeps the eyes irritated and protracts the cure—as soon as possible. Blisters and counter-irritants of all kinds should be strictly avoided, as doing no good and increasing the eruption of the face. Should the disease of the cornea assume the form of deep ulceration and hypopium, the energetic use of atropin, the compressive bandage, combined with paracentesis corneæ once a-day, have yielded far better results than any other means that I have tried. The paracentesis often checks the progress of the ulcer immediately, and prevents perforation; or if it does not prevent perforation, it limits the lateral extension of the ulcer, and thus helps to save the cornea. I generally puncture the cornea once a-day, under chloroform if necessary, for several days, till the hypopium and extension of the ulcer are relieved. I puncture the cornea to evacuate the aqueous humor and relieve tension, without regard to the pus. After the paracentesis, the pus or lymph is rapidly absorbed. I select the outer part of the cornea for the operation, simply because it is most convenient. I cannot too highly recommend this little operation in all cases of hypopium keratitis, as recently described by Roser, Von Graefe, Weber of Darmstadt, and others. Where there is a distinct abscess of the cornea, Weber's recommendation to puncture through the collection of pus is sometimes preferable, but must be done with great caution, to prevent the too sudden evacuation of the aqueous humor. He enters the needle at the most dependent point of the pustule, and passes it obliquely through, so as to enter the anterior chamber at the upper part. The aqueous fluid thus washes out the abscess as it escapes, and the further subsidence of the pus between the lamellæ is prevented. As a rule, I much prefer puncturing away from the seat of the ulcer.

I would say, for the information of any who may wish to get a good sample of the brown salve, that it can be ordered in any quantity of Suire & Co., druggists, N.-W. corner 4th and Vine streets, Cincinnati. I will not consume space in the discussing of the merits of other forms of mercurial salves, powdered calomel, etc., in these affections. They are all useful when used with discrimination, but in my practice very much inferior to the brown salve. The use of a seton in the temple, in phlyctenular corneitis, as recommended lately in the Ophthalmic Hospital Reports (London), Vol. IV., part iii, by Watson, I have never tried, and probably never will. Dr. Pagenstecher, of Wiesbaden, in his Klinische Beobachtungen, etc., 1861, recommends a salve of the amorphous yellow oxide of mercury very highly in the same cases. I tried it some years ago, but found it was very much more irritating than the brown salve and less efficacious. In the *Ophthalmic Review* of July, 1865, is an excellent article from the pen of Dr. Pagenstecher, on the value of yellow oxide of mercury ointment, and the method of preparation and using. I have no doubt but he gives a candid and well-formed opinion of its efficiency; but a comparative trial of the two preparations has led me to adhere to the brown salve as much preferable.—*N. Y. Medical Record.*

